# The experimental design of postmortem studies: the effect size and statistical power

**DOI:** 10.1007/s12024-016-9793-x

**Published:** 2016-07-13

**Authors:** Joris Meurs

**Affiliations:** Department of BioAnalytical Chemistry, VU University Amsterdam, De Boelelaan, 1081 HV Amsterdam, The Netherlands

**Keywords:** Postmortem research, Sample size, Experimental design, Significance, Power, Effect size

## Abstract

**Purpose:**

The aim is of this study was to show the poor statistical power of postmortem studies. Further, this study aimed to find an estimate of the effect size for postmortem studies in order to show the importance of this parameter. This can be an aid in performing power analysis to determine a minimal sample size.

**Methods:**

GPower was used to perform calculations on sample size, effect size, and statistical power. The minimal significance (*α*) and statistical power (1 − *β*) were set at 0.05 and 0.80 respectively. Calculations were performed for two groups (Student’s *t*-distribution) and multiple groups (one-way ANOVA; *F*-distribution).

**Results:**

In this study, an average effect size of 0.46 was found (*n* = 22; SD = 0.30). Using this value to calculate the statistical power of another group of postmortem studies (*n* = 5) revealed that the average statistical power of these studies was poor (1 − *β* < 0.80).

**Conclusion:**

The probability of a type-II error in postmortem studies is considerable. In order to enhance statistical power of postmortem studies, power analysis should be performed in which the effect size found in this study can be used as a guideline.

## Introduction

**Fig. 1:**
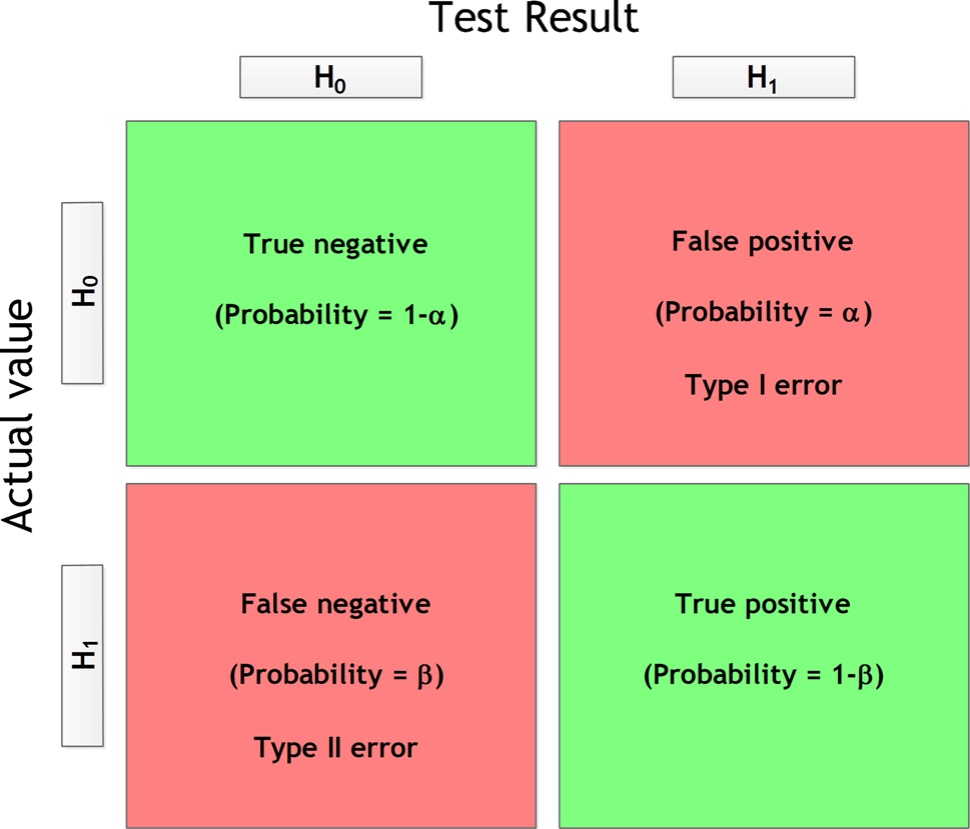
The confusion matrix of accepting or rejecting the null hypothesis (H_0_) or the alternative hypothesis (H_1_)

Prior to conducting research, several considerations have to be made. For example, the required sample size has to be determined [1]. Commonly, this is done by performing a so-called power analysis [1, 2]. In a power analysis, the sample size is calculated by using four parameters: significance (*α*), statistical power (1 − *β*), variance (*σ*^2^), and effect size (*d*) [1, 3]. A description and the effect on the sample size of each of these parameters is shown in Table 1. In order to emphasize the effect of *α* and 1 − *β*, the confusion matrix is shown in Fig. 1. Despite *α* and 1 − *β* being mostly straightforward values, determining *σ*^2^ and *d* is rather difficult [1]. In case two independent means are present, Cohen set values of *d* at 0.20, 0.50, and 0.80 which represent a small, medium, or large effect size respectively [1]. The effect sizes in case multiple means (multiple groups) are present have been set at 0.10, 0.25, and 0.40, which represent a small, medium, or large effect size respectively. According to Cohen, his set medium value for *d* represents “an effect likely to be visible to the naked eye” [1]. For instance, this can be a change in decomposition stage of a cadaver. In quantitative research this visible effect could be, for example, a significant change in concentration of a certain analyte in a postmortem sample. Nevertheless, for inexperienced individuals it still remains unclear what the actual meaning of *d* is. The effect size is defined as the absolute difference between two independent means and the within-sample standard deviation [1, 4]. In other words, how much does a certain situation (e.g., a qualitative or quantitative experiment) differ from reality? Moreover, for calculating *d* values the independent means (*μ*_*a*_; *μ*_*a*_) and the within-sample standard deviation (*σ*) have to be estimated [1]. Hence, the resulting *d* will be a rather subjective value. To solve this problem, a pilot study can be performed and a sample standard deviation can be used for calculating the effect size [3, 4]. However, pilot studies lack statistical power [5]. Hence, performing a pilot study is not desirable.

**Table 1:** Description and effect of parameters on sample size

Parameter	Description	Effect on sample size
Alpha (*α*)	The probability of falsely rejecting the null hypothesis (H_0_) (i.e., false positive result or type I error)^a^ [5]	The lower *α*, the higher the sample size
Beta (*β*)	The probability of falsely accepting the null hypothesis (H_0_) (i.e., false negative result or type II error)^a^ [5]	The lower *β*, the higher the sample size
Power (1 − *β*)	The probability of correctly rejecting the null hypothesis (H_0_)^a^ [5]	The higher 1 − *β*, the higher the sample size
Effect size (*d* or *f*)	Degree of deviation of an experimental situation compared to an actual situation (i.e., how much does an experiment deviate from reality) [5]	The higher *d* or *f*, the lower the sample size
Variance (*σ* ^2^)	Expression of the spreading of data around a mean value [5]	The higher *σ* ^2^, the higher the sample size
Noncentrality parameter (*λ*)	Degree of deviation from the original distribution [15]	*λ* = 0: original distribution *λ* > 0: increasing noncentrality

^a^See Fig. 1 for a graphical explanation

It is observed that in postmortem research the sample size is variable. For instance, the sample size can be as low as nine [6] or as high as 57,903 [7]. Low availability of samples or legal restrictions can be a reason for small sample sizes. Although, parameters like the statistical power should still be taken into account despite these limitations. No discussion on the sample size used or the statistical power reached is seen in most publications. Hence, the probability is of false-negative results cannot be derived from the data that is shown [4]. Therefore, the aim of this paper is to show how a minimal sample size can be estimated without a priori knowledge on the standard deviation to ensure sufficient statistical power. Furthermore, the poor statistical power of postmortem studies will be shown.

## Calculation of the sample size in general cases

### Two independent means (Student’s *t* test)

To calculate the sample size (*n*) in order to compare two independent means, Eq. 1 has to be solved [4].

1 $$ n = \left( {\left( {z\alpha /2 + z\beta } \right)\frac{\sigma }{\delta }} \right)^{2} $$

where, *z* is the corresponding *z* score for values of *α* and *β* and *δ* is defined as the absolute difference between the experimental mean (*μ*_*a*_) and the control mean (*μ*_*b*_) (Eq. 2).

2 $$ \delta = \left| {\mu_{a} - \mu_{b} } \right| $$

To calculate the *z* score, values for *α* were set at 0.05 and 0.01 respectively. Likewise, values for *β* were set at 0.20, 0.10, and 0.05 respectively. All obtained values are shown below in matrix *Z*. Column 1 and 2 contain the values for significance levels of 0.05 and 0.01. Values for *β* decrease going down the rows.

3 $$ Z = \left[ {\begin{array}{*{20}c} {\alpha = 0.05;\beta = 0.20} & {\alpha = 0.01;\beta = 0.20} \\ {\alpha = 0.05;\beta = 0.10} & {\alpha = 0.01;\beta = 0.10} \\ {\alpha = 0.05;\beta = 0.05} & {\alpha = 0.01;\beta = 0.05} \\ \end{array} } \right] = \left[ {\begin{array}{*{20}c} {7.849} & {11.6790} \\ {10.5074} & {14.8794} \\ {12.9947} & {17.8142} \\ \end{array} } \right] $$

**Fig. 2:**
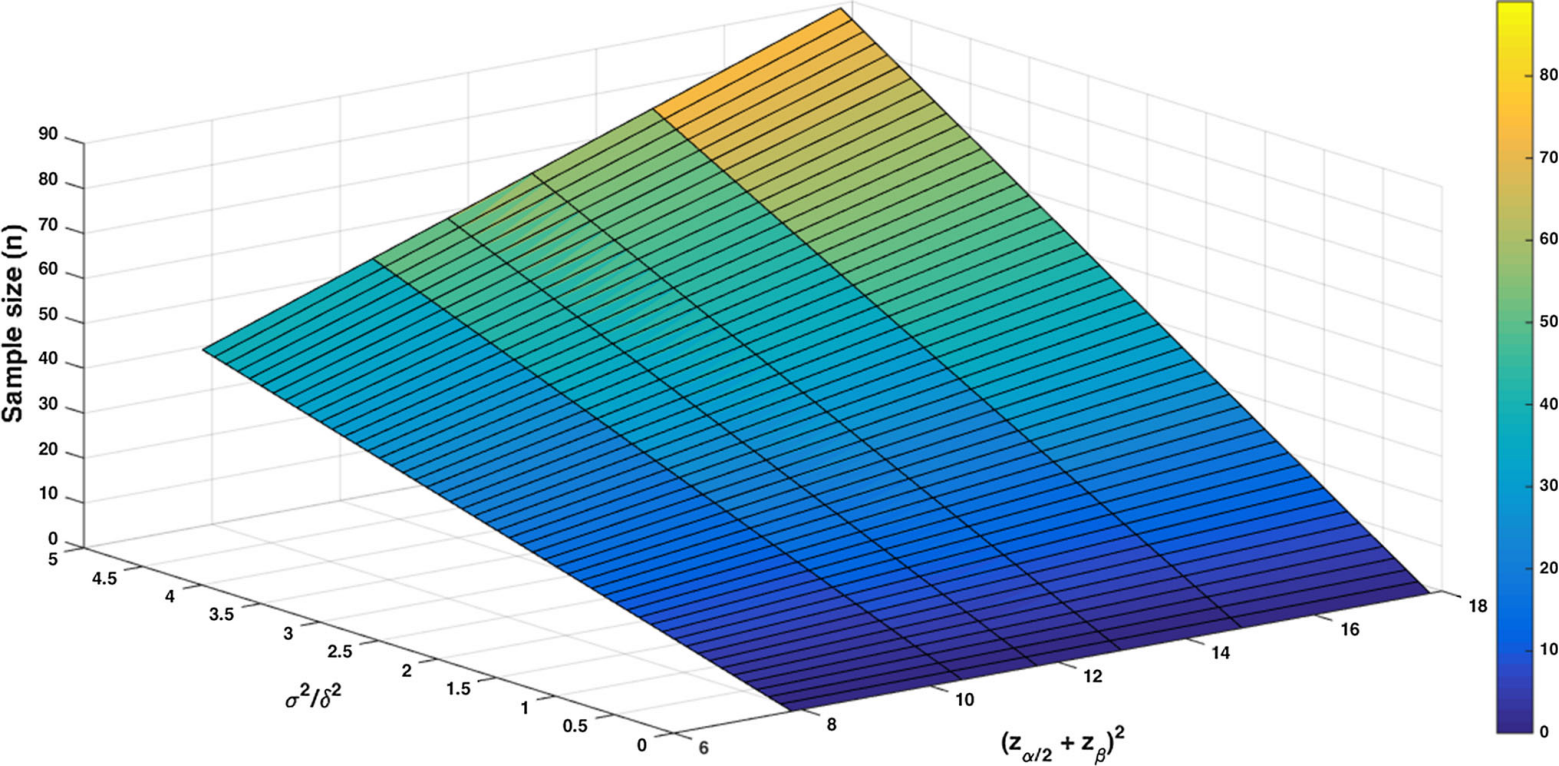
Influence of (*z*_*α*_ + *z*_*β*_)^2^ and *σ* ^2^/*δ* ^2^ on the sample size

**Fig. 3:**
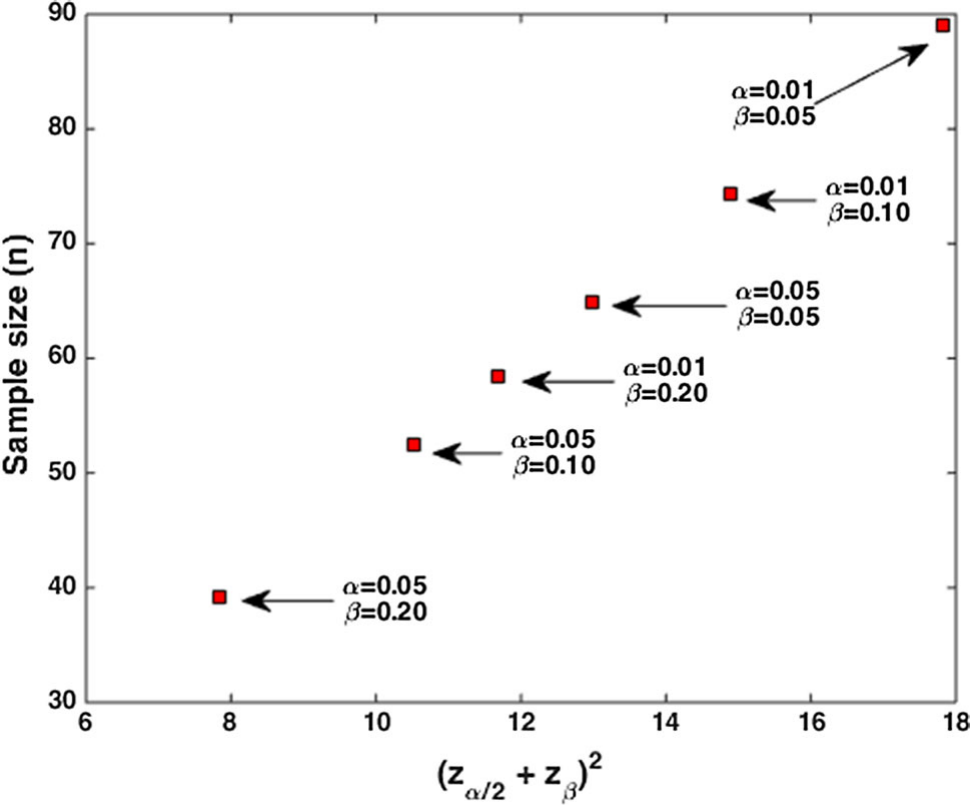
Sample size for different values of *α* and *β* at maximum *σ* ^2^/*δ* ^2^

According to Cohen, the effect size is considered as small, medium, or large at values of 0.20, 0.50, and 0.80 respectively [1]. Since *σ*/*δ* is inversely related to the effect size, *σ*/*δ***-**values of 5, 2, and 1.25 can be considered as large, medium, and small respectively. Therefore, values for the ratio *σ*^2^/*δ*^2^ were set from 0 to 5. With these values, the corresponding sample size (*n*) was calculated (Fig. 2). To obtain a reasonable estimate for the minimal sample size, for all combinations of *α* and *β* the sample size was calculated at the maximum ratio of *σ*^2^/*δ*^2^. These values are shown in Table 2 and Fig. 3.

**Table 2:** Overview of sample size in case of two independent means (two groups) at common values of *α* and *β* at high value of *σ* ^2^/*δ* ^2^

*α*	*β*	Power (1 − *β*)	Sample size (*n*)^a^
0.05	0.20	0.80	39
0.05	0.10	0.90	52
0.05	0.05	0.95	65
0.01	0.20	0.80	58
0.01	0.10	0.90	74
0.01	0.05	0.95	89

Sample size calculated for equal group sizes

^a^Values for *n* are rounded to the nearest integer

### Multiple means (ANOVA)

In case of multiple means, the sample size should be determined by using ANOVA. The effect size (*f*) is then expressed as follows (Eq. 4) [1, 8]:

4 $$ f = \frac{{\sigma_{m} }}{\sigma } $$

Accordingly, the total sample size is calculated by using Eq. 5, in which *N* is the total sample size and *λ* is the noncentrality parameter [9, 10]. This noncentrality parameter is about 1.5 for *α* = 0.01 when *β* = 0.20 and about 1 for *α* = 0.05 when *β* = 0.20 [10].

5 $$ N = \frac{\lambda }{{f^{2} }} $$

**Fig. 4:**
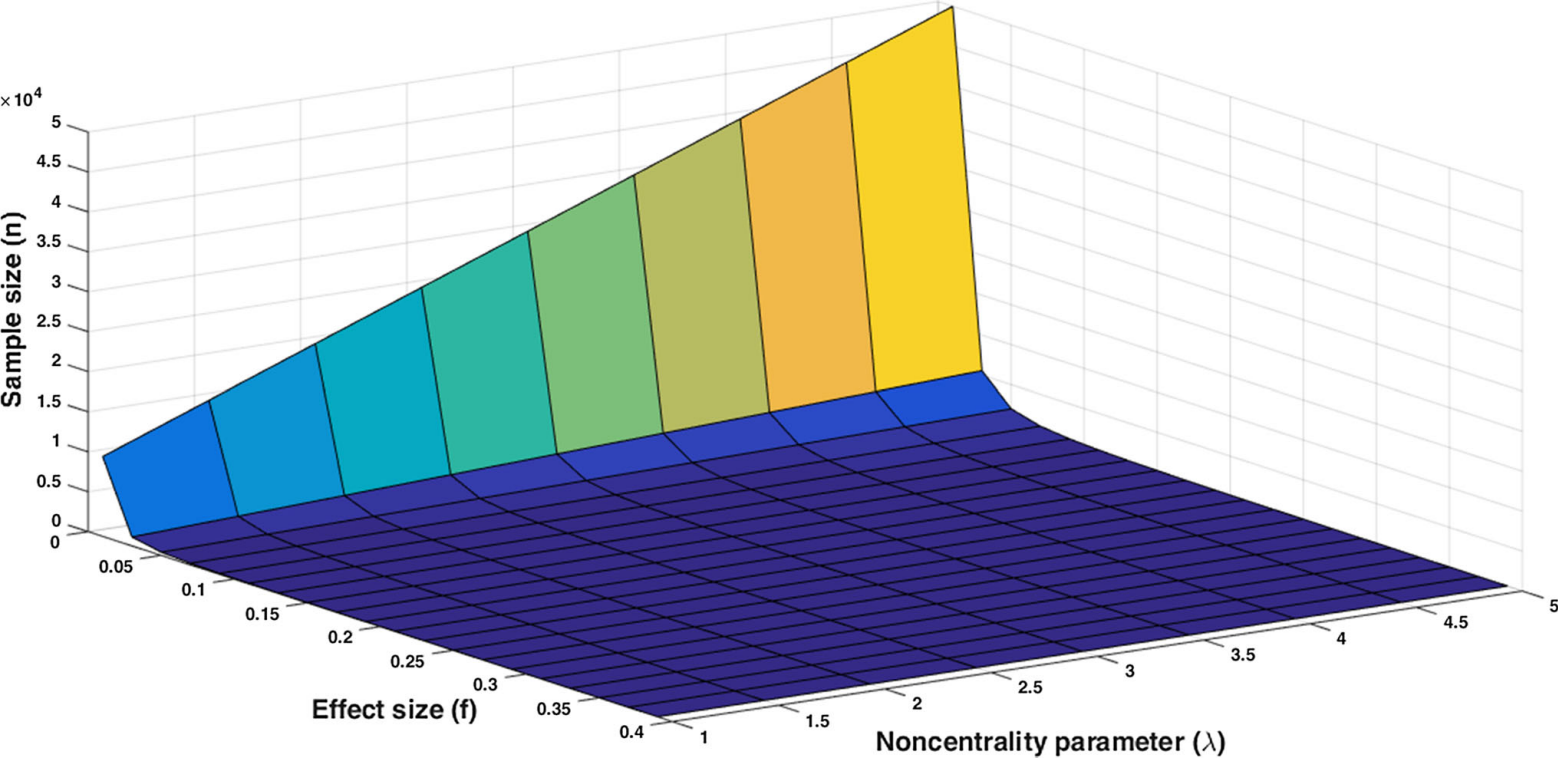
Influence of *f* and *λ* on the sample size

For the one-way ANOVA model, Cohen’s values of 0.10, 0.25, and 0.40 were used to calculate the minimal sample size at significance levels of 0.05 and 0.01 respectively. These results are shown in Table 3 and Fig. 4.

**Table 3:** Overview of sample size in case of multiple means (multiple groups) at common values of *α* and *f* (*β* = 0.20)

*α*	*f*	*N* (*k* = 3)	*N* (*k* = 4)	*N* (*k* = 5)	*N* (*k* = 8)	*N* (*k* = 10)
0.01	0.10	1395	1552	1685	1992	2160
0.01	0.25	228	256	275	328	360
0.01	0.40	93	104	115	136	150
0.05	0.10	969	1096	1200	1448	1580
0.05	0.25	159	180	200	240	260
0.05	0.40	66	76	80	104	110

*k*, group size; values are calculated in GPower [8]

## Statistical power and effect size of postmortem studies

In order to show the poor statistical power of postmortem studies, a number of studies were selected for post hoc testing on the sample size in order to determine the achieved power. For calculations GPower was used [8]. First, the effect size for a number of postmortem studies (*n* = 22) was calculated. This data is shown in Table 4. Significance level and statistical power were set at 0.05 and 0.80 respectively. A mean effect size of 0.46 (SD = 0.30) was obtained.

**Table 4:** Effect size calculation for a number of postmortem studies

References	Sample size (*n*)	Number of groups	Effect size
Rognum et al. [16]	87	4	0.36
Sato et al. [17]	18	6	1.05
Singh et al. [18]	474	9	0.18
Singh et al. [19]	1026	15	0.13
Wehnet et al. [20]	128	45	0.55
Mihailovic et al. [21]	320	10	0.22
Lemaire et al. [22]	30	4	0.65
Laruelle et al. [23]	34	4	0.60
Pelander et al. [24]	50	2	0.40
Vujanić et al. [25]	540	6	0.15
Krap et al. [26]	10	2	1.01
Li et al. [27]	283	4	0.20
Zhu et al. [28]	405	5	0.17
Koopmanschap et al. [29]	117	3	0.29
Zhu et al. [30]	234	4	0.22
Huang et al. [31]	90	10	0.43
Zheng et al. [32]	111	37	0.56
Li et al. [33]	12	2	0.90
Rognum et al. [34]	32	3	0.58
Maeda et al. [35]	140	4	0.28
Zhu et al. [36]	409	15	0.21
Frere et al. [37]	10	2	1.01
	207.3 ± 246.5	8.9 ± 11.1	0.46 ± 0.30

*α* = 0.05; *β* = 0.20; * *p* < 0.05; values are calculated in GPower [8]

This effect size was used to calculate the achieved statistical power of another group of postmortem studies (*n* = 5). A priori, the significance was set at 0.05. The results are shown in Table 5. Only for the studies of Mao et al. [11] and Laiho and Penttilä [12] was the achieved statistical power sufficient (i.e., a value greater than 0.80). In all other cases, the statistical power was less than 0.80, which means there is a reasonable probability of a type-II error. Despite these low power values, the risk of false-negative results are not discussed. An example of a false-negative result is that no significant difference is found in concentration while in fact there is a significant difference. In other words, the null hypothesis (H_0_) has been falsely rejected.

**Table 5:** Post hoc testing performed on a number of postmortem studies (*f* = 0.46)

References	Sample size (*n*)	Number of groups	Achieved power (1 − *β*)
Mao et al. [11]	160	2	0.99
Moriya and Hashimoto [38]	6	2	0.14
Mao et al. [39]	48	6	0.62
Querido and Pillay [40]	36	6	0.46
Laiho and Pentillä [12]^a^	116	8	0.96
	73.2 ± 63.0	4.80 ± 2.68	0.63 ± 0.36

Achieved power was calculated using GPower [8]. Post hoc testing was performed using a one-way ANOVA model with fixed effects

^a^Groups were not divided into equal numbers

## Discussion and conclusion

Power analysis can be a useful tool in determining the sample size needed for qualitative and quantitative postmortem experiments. Examples of postmortem qualitative and quantitative research are determining the degree of decomposition [13] and measuring postmortem vitreous potassium [14]. However, in order to calculate the sample size, values have to be set subjectively.

That can be a cause of choosing a random sample size in postmortem research. Sample size determination and achieved statistical power are rarely discussed in postmortem studies. However, it is important to discuss these parameters in order to establish the reliability of the obtained results.

This study is the first to demonstrate that postmortem studies lack statistical power. In order to achieve sufficient power, Tables 2 and 3 can be used for obtaining a minimal sample size for common values of significance and statistical power. However, it should always be checked a posteriori if the set levels of power and significance are achieved by performing a post hoc test. Nevertheless, Tables 2 and 3 can serve as a useful tool in estimating a minimal sample size that would provide sufficient statistical power for postmortem studies.

Additionally, for the first time an estimate of the effect size (*f* = 0.46; SD = 0.30) has been shown for postmortem studies. Besides Tables 2 and 3, this number can be used as an estimate for the effect size in power analysis.

## Key Points

1. An effect size has been estimated for postmortem studies.
2. The statistical power of postmortem studies is poor.
3. Power analysis should be performed in order to enhance statistical power of postmortem studies.
